# Human in vitro induced T regulatory cells and memory T cells share common demethylation of specific FOXP3 promoter region

**DOI:** 10.1186/s13601-015-0079-2

**Published:** 2015-10-20

**Authors:** Philippe Bégin, Janika Schulze, Udo Baron, Sven Olek, Rebecca N. Bauer, Laura Passerini, Rosa Baccheta, Kari C. Nadeau

**Affiliations:** Division of Allergy, Immunology and Rheumatology, Department of Pediatrics, Stanford University, Stanford, CA USA; Epiontis, Berlin, Germany; San Raffaele Telethon Institute for Gene Therapy (HSR-TIGET), Division of Regenerative Medicine, Stem Cells and Gene Therapy, San Raffaele Scientific Institute, Milan, Italy; Division of Allergy, Department of Pediatrics, CHU Sainte-Justine, Montreal, Canada

**Keywords:** Treg, Epigenetic, TSDR, FOXP3, Methylation, Promoter, Assay, IPDR, Induced

## Abstract

**Background:**

The FOXP3 gene is the master regulator for T regulatory cells and is under tight DNA methylation control at the Treg specific demethylated region (TSDR) in its first intron. This said, methylation of its promoter region, the significance of which is unknown, has also been associated with various immune-related disease states such as asthma, food allergy, auto-immunity and cancer. Here, we used induced T regulatory cells (iTreg) as a target cell population to identify candidate hypomethylated CpG sites in the FOXP3 gene promoter to design a DNA methylation quantitative assay for this region.

**Findings:**

Three CpG sites at the promoter region showed clear demethylation pattern associated with high FOXP3 expression after activation in presence of TGFβ and were selected as primary targets to design methylation-dependent RT-PCR primers and probes. We then examined the methylation of this ‘inducible-promoter-demethylated-region’ (IPDR) in various FOXP3+ T cell subsets. Both naïve and memory thymic-derived Treg cells were found to be fully demethylated at both the IPDR and TSDR. Interestingly, in addition to iTregs, both CD25− and CD25^lo^ conventional memory CD4+CD45RA− T cells displayed a high fraction of IPDR demethylated cells in absence of TSDR demethylation.

**Conclusion:**

This implies that the fraction of memory T cells should be taken in account when interpreting FOXP3 promoter methylation results from clinical studies. This approach, which is available for testing in clinical samples could have diagnostic and prognostic value in patients with immune or auto-inflammatory diseases.

## Findings

The FOXP3 gene plays a key role in regulating the immune response and its expression occurs under tight epigenetic control [1]. Notably, DNA demethylation of the ‘Treg-specific-demethylated-region’ (TSDR), located on the 2nd conserved non-coding sequence of FOXP3 (CNS2), is a *sine qua non* modification found in thymic-derived T regulatory cells (tTregs—formerly known as nTregs) [2]. TSDR demethylation results in stable and high FOXP3 expression and suppressive function in tTregs. Using a modified RT-PCR approach, the amount of TSDR hypomethylation can be precisely measured from whole DNA providing a highly accurate quantitative biomarker to identify tTregs within whole blood, PBMCs or tissue samples [3].

This said, epigenetic studies using pyrosequencing have also associated other loci on the FOXP3 gene, such as its promoter region, with various immune-related disease states such as asthma [4], food allergy [5], auto-immunity [6, 7] and cancer [8]. Furthermore, the FOXP3+ T cell compartment comprises other cell subsets with methylated TSDR which have variable suppressive function [9] and FOXP3 can be transiently expressed by any non-suppressive T conventional (Tconv) cells upon activation [10]. In mice, knocking-out the CNS1 results in a loss of peripheral tolerance with allergy and maternal-fetal conflict despite functional tTregs showing the importance of other control regions on FOXP3 and of non-tTreg FOXP3+ subsets to regulate the immune response [11]. We hypothesized that FOXP3+ subset-specific DNA methylation sites could be identified outside of the TSDR and used additively to design a more comprehensive, high-throughput assay to characterize the FOXP3+ T cell population in clinical samples. Characterizing the FOXP3+ T cell populations could have a high impact in monitoring and treating many diseases, including those diseases with autoimmunity, cancer, and allergy.

Given their potential clinical relevance and lack of available markers, we performed studies using induced Tregs (iTreg) as a target cell population to identify candidate hypomethylated CpG sites. iTregs are typically generated in vitro from naïve T cells with TCR-mediated stimulation in the presence of tumor growth factor (TGF)-β and interleukin (IL)-2. The expression of FOXP3 from the resulting iTreg population is very high but these cells remain fully methylated at the TSDR [1]. In humans, their suppressive function is not reliable although the addition of retinoic acid to the induction protocol has been reported to increase the stability of FOXP3 expression and of the suppressive function [12, 13].

**Fig. 1:**
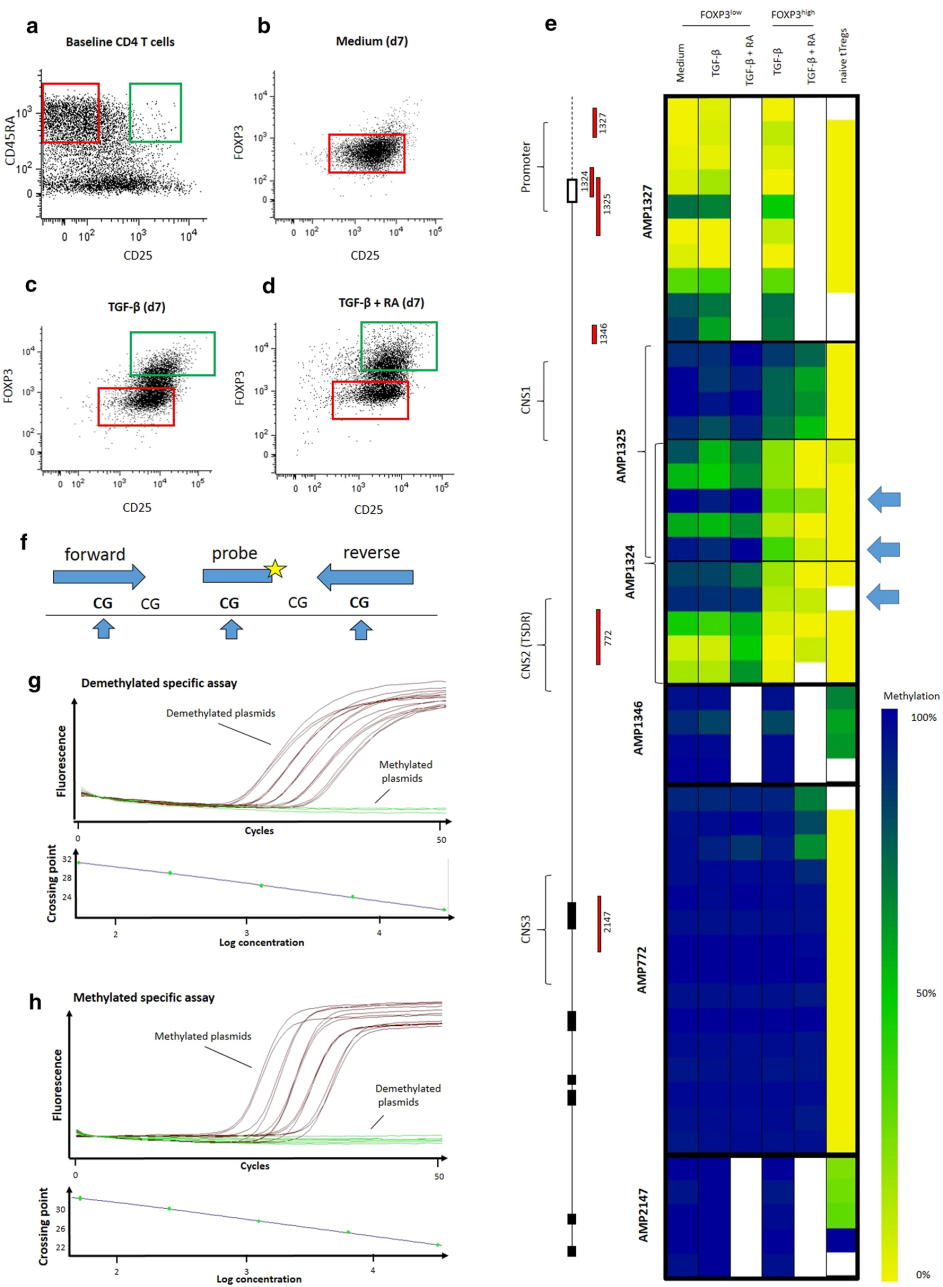
FOXP3 promoter RT-PCR assay design. Naïve T cells (CD4+CD45RA+CD62L+CD25−) were sorted from CD4 enriched buffy coats (**a**, *red gate*) and put in culture with IL-2 and CD3/CD28 stimulation in medium alone (**b**), in the presence of TGF-β (**c**) or both TGF-β and retinoic acid (**d**). At day 7, cells were sorted based on FOXP3 expression. FOXP3^high^ (*green gates*) and FOXP3^low^ samples (*red gates*), as well as baseline naïve tTreg controls (**a**, *green gate*) were pyrosequenced at the promoter and three conserved non-coding sequence regions of the FOXP3 gene (**e**). Promoter CpG sites at positions −126, −77 and −56 from transcription start site showed the greatest differential methylation in FOXP3^high^ TGF-induced T cells (iTregs) (indicated with *arrows*). These sites were selected to design primers and RT-PCR probes specific for their methylation status (**f**). Specificity of methylated and unmethylated RT-PCR primers and probes was validated using unmethylated (**g**) and methylated (**h**) plasmid standards dilutions at 31,250, 6250, 1250, 250 and 50 copy numbers per reaction

Briefly, naïve T cells were cell-sorted (BD FACSAria II^®^) from healthy control peripheral blood on consented adult blood bank donors as CD4+CD45RA+CD62L+CD25− and put in culture in AIM V medium (Life Technologies^®^) with 100 U/mL IL-2 (BD Pharmingen^®^), 10 ng/mL TGF-β1 (R&D Systems^®^), and/or 100 nM ATRA (Sigma-Aldrich^®^) and anti-CD3/CD28 mAbs Dynabeads (Life Technologies^®^) at a bead:cell ratio of 1:20 (Fig. 1a). Beads were removed with magnet at day 4 and cells were washed and re-plated with fresh medium with IL-2 at 100 UI/L. Cells were fixed at day 7 and sorted based on their intracellular expression of FOXP3 (Fig. 1b–d). DNA was extracted and bisulfite treated for pyrosequencing of the FOXP3 promoter and three conserved noncoding sequences regions (Fig. 1e).

Interestingly, three CpG sites at the promoter regions (at positions −126, −77 and −56 from transcription start site) showed clear demethylation pattern associated with high FOXP3 expression after activation in presence of TGFβ (Fig. 1e). There were no changes in demethylation of TSDR or other CNS regions. Addition of retinoic acid to TGFβ did not impact the demethylation of any CpG sites in the FOXP3 locus compared to TGFβ alone. The three CpG sites in the promoter region were selected as primary targets to design primers and probes to quantify the methylation of this ‘inducible-promoter-demethylated-region’ (IPDR) using the same method established for the TSDR (Fig. 1f) [3, 14].

**Fig. 2:**
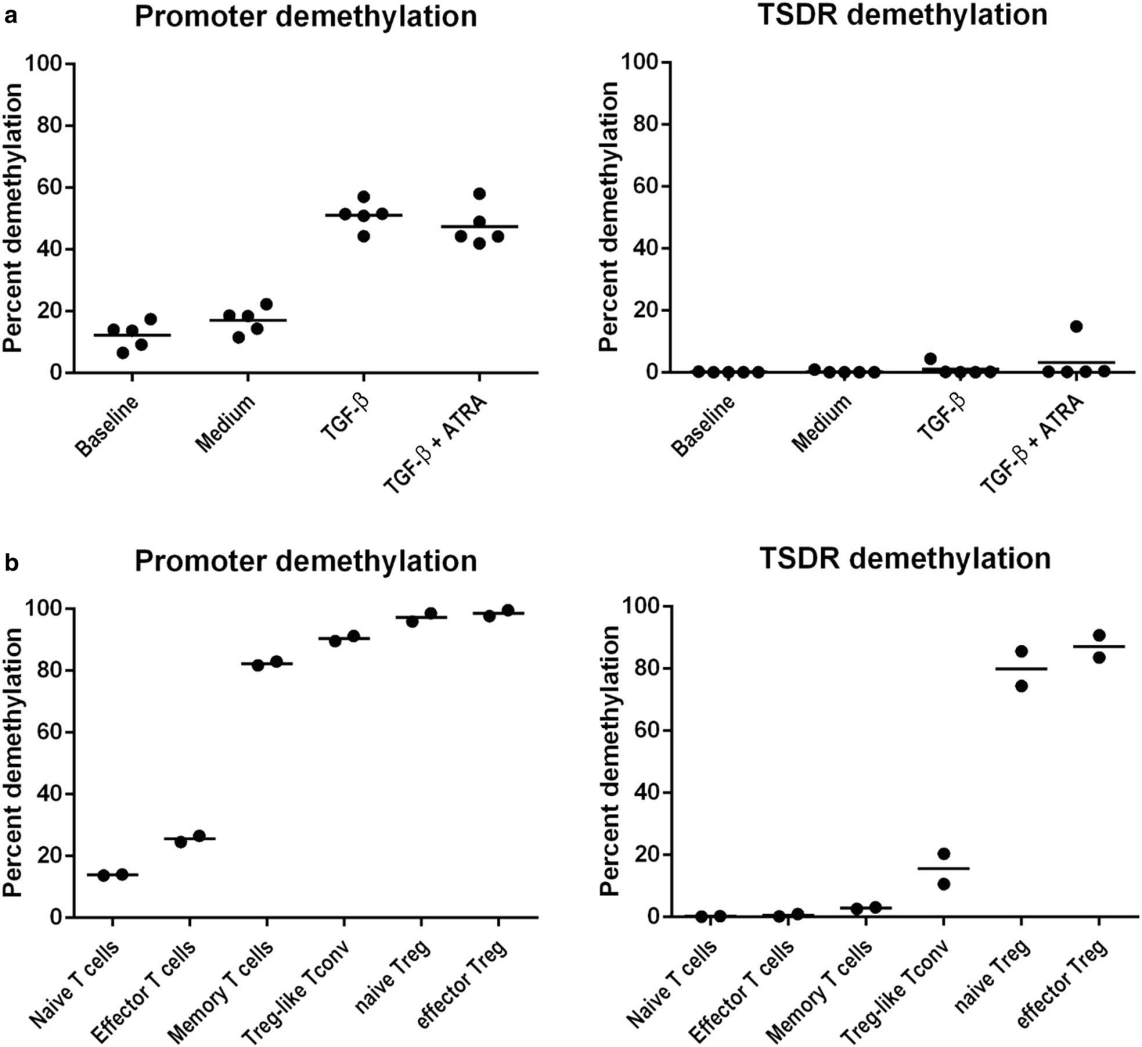
RT-PCR quantification of FOXP3 promoter and TSDR demethylation in CD4 T cell subpopulations. Culture of naïve CD4 T cells for 7 days with IL-2 and CD3/CD28 stimulation induced the demethylation of the FOXP3 promoter in the presence of TGF-β and/or retinoic acid (iTregs) but not with medium alone (**a**). FOXP3 promoter demethylation in absence of TSDR demethylation was also found to be a hallmark of CD45RA−CD25−CD4+ memory T cells and CD45RA−CD25^low^CD4+ “Treg-like” T conventional cells, but not of CD45RA+CD62L+CD4+ naïve or CD45RA+CD62L−CD4+ effector T cells (**b**)

The specificity and sensitivity of the assay was first confirmed by using methylated and demethylated synthetic plasmid sequences (Fig. 1g, h) and then validated in cell populations with high methylation of the IPDR, as tested by pyrosequencing (i.e. granulocytes, monocytes, B cells and NK cells). To validate its quantitative value in samples, the IPDR assay was performed on whole T cells from an iTreg induction culture at baseline and day 7. A low percentage of naïve T cells showed IPDR demethylation at baseline. At day 7, in the presence of TGF-β, the fraction of IPDR demethylation was increased and the increment was correlated with the increased frequency of cells expressing high levels of FOXP3 protein (Fig. 2a).

To investigate the distribution of IPDR demethylation in FOXP3+ T cell subsets we applied the assay to fresh peripheral T cell populations based on CD25 and CD45RA expression according to previously described classification [1]. Both naïve and memory tTreg cells (Fig. 2a) were found to be fully demethylated at both the IPDR and TSDR. Interestingly, both CD25− and CD25^lo^ conventional memory CD4+ T cells displayed a high fraction of IPDR demethylated cells (Fig. 2b). Thus, while TSDR demethylation is required for sustained high expression of FOXP3 in tTregs, IPDR demethylation is associated with a wide range of (possibly transient) FOXP3 protein expression in antigen-experienced T cells, ranging from negative to low protein expression in memory T cells, to high expression in iTregs. The high expression of FOXP3 protein in iTreg could be due to the additional effect of epigenetic modifications such as histone modifications, as suggested in mice [15]. Inversely, in non-activated CD25^−/lo^ memory T cells, the FOXP3 protein could be down-regulated at the post-translational level by STUB1-mediated ubiquitination and proteasomal degradation of the protein, which is induced by pro-inflammatory stimuli (IL-1, IL-6, LPS) [16].

Such an ability to express and modulate low levels of FOXP3 with IPDR demethylation could be important for the homeostatic control of the antigen-experienced T cell population. Knocking down FOXP3 in non-regulatory T cells with siRNA demonstrated FOXP3 expression is critical for limiting cellular proliferation and cytokine production (intrinsic regulation) [10].

This said, the variable demethylation of the IPDR in T cell subsets means that it cannot be used to quantify subsets from unfractioned blood like the TSDR assay. Controlling for CD45 expression in CD3 positive T cells may help improve test interpretation on mixed population sample but it is insufficient to account for all the inter-individual variability (data not shown) which may also stem demethylation of IPDR in effector (<30 %), naïve T cells (<20 %) which despite being low does contribute to the whole amount of demethylated DNA. This raises questions with regards to previous associations of FOXP3 promoter demethylation with immune disease states which were based on unfractioned blood analyses, which may need to be revisited by measuring IPDR demethylation in sorted specific T cell subsets. Further in depth analyses of IPDR demethylation in T cell subsets in large cohorts will also be needed to provide reference values for such analyses. Better characterization of IPDR demethylation in various subsets may eventually provide surface markers that will allow its interpretation in unfractioned samples.

In summary, we have identified an *inducible promoter demethylated region* (IPDR) of FOXP3 and designed a quantitative assay to accurately measure its methylation which is available for testing in clinical samples. Since IPDR demethylation in absence of TSDR demethylation is a feature of both iTregs and memory T cells, its use will minimally require taking in account the fraction of cell subsets in a given mixed sample, or ideally, pre-sorting of cell subsets prior to analysis. This approach could have diagnostic and prognostic value in patients with immune or auto-inflammatory diseases.

## Abbreviations

ATRA: all-trans retinoic acid
CNS: conserved non-coding sequence
IL-: interleukin
IPDR: inducible promoter demethylated region
iTreg: inducible T regulatory cell
LPS: lipopolysaccharide
PBMC: peripheral blood mononucleated cell
T conv: non-suppressive conventional T cell
TGF-β: tumor growth factor beta
TSDR: Treg-specific demethylated region
tTreg: thymic-derived T regulatory cell
